# A novel metabolomic approach used for the comparison of *Staphylococcus aureus* planktonic cells and biofilm samples

**DOI:** 10.1007/s11306-016-1002-0

**Published:** 2016-03-08

**Authors:** Laurence H. Stipetic, Matthew J. Dalby, Robert L. Davies, Fraser R. Morton, Gordon Ramage, Karl E. V. Burgess

**Affiliations:** Institute of Infection, Immunity and Inflammation, College of Medical, Veterinary and Life Sciences, The University of Glasgow, Glasgow, UK; Glasgow Polyomics, Wolfson Wohl Cancer Research Centre, The University of Glasgow, Garscube Estate, Bearsden, Scotland G61 1QH UK; Institute of Molecular Cell and Systems Biology, The University of Glasgow, Glasgow, UK; Infection and Immunity Research Group, Glasgow Dental School, School of Medicine, College of Medical, Veterinary and Life Sciences, The University of Glasgow, Glasgow, UK

**Keywords:** Metabolomics, Metabolite extraction, Biofilms, *S. aureus*

## Abstract

**Introduction:**

Bacterial cell characteristics change significantly during differentiation between planktonic and biofilm states. While established methods exist to detect and identify transcriptional and proteomic changes, metabolic fluctuations that distinguish these developmental stages have been less amenable to investigation.

**Objectives:**

The objectives of the study were to develop a robust reproducible sample preparation methodology for high throughput biofilm analysis and to determine differences between Staphylococcus aureus in planktonic and biofilm states.

**Methods:**

The method uses bead beating in a chloroform/methanol/water extraction solvent to both disrupt cells and quench metabolism. Verification of the method was performed using liquid-chromatography-mass spectrometry. Raw mass-spectrometry data was analysed using an in-house bioinformatics pipe-line incorporating XCMS, MzMatch and in-house R-scripts, with identifications matched to internal standards and metabolite data-base entries.

**Results:**

We have demonstrated a novel mechanical bead beating method that has been optimised for the extraction of the metabolome from cells of a clinical Staphylococcus aureus strain existing in a planktonic or biofilm state. This high-throughput method is fast and reproducible, allowing for direct comparison between different bacterial growth states. Significant changes in arginine biosynthesis were identified between the two cell populations.

**Conclusions:**

The method described herein represents a valuable tool in studying microbial biochemistry at a molecular level. While the methodology is generally applicable to the lysis and extraction of metabolites from Gram positive bacteria, it is particularly applicable to biofilms. Bacteria that exist as a biofilm are shown to be highly distinct metabolically from their ‘free living’ counterparts, thus highlighting the need to study microbes in different growth states. Metabolomics can successfully distinguish between a planktonic and biofilm growth state. Importantly, this study design, incorporating metabolomics, could be optimised for studying the effects of antimicrobials and drug modes of action, potentially providing explanations and mechanisms of antibiotic resistance and to help devise new antimicrobials.

**Electronic supplementary material:**

The online version of this article (doi:10.1007/s11306-016-1002-0) contains supplementary material, which is available to authorized users.

## Introduction

Metabolomics aims to measure comprehensively the metabolic profile of a system (Fiehn 2002). The term metabolomics was first coined in 1998 (Oliver et al. 1998), although the concept of ‘metabolic profiling’ can be dated to the 1970s (Horning and Horning 1971). Metabolomics provides a ‘snap-shot’ of cell metabolism and can highlight discrete changes in metabolic pathways and the abundance of biological small molecule metabolite intermediates. Using untargeted metabolomics, chemical effects across the gamut of metabolic processes can be studied providing greater understanding of the biological responses from single cells to the complex system/organism level (Nicholson and Lindon 2008).

Metabolomic techniques have been applied to bacterial cells (Tang 2011; Liebeke et al. 2012), although not without significant hurdles. Current microbial extraction and metabolic quenching methods have a number of associated problems (de Koning and van Dam 1992; Bolten et al. 2007). Often, metabolite degradation or inadequate quenching of metabolism during extraction, media contamination, or failure to lyse the bacterial cell, can lead to artefactual and/or variable results (Bolten et al. 2007). In addition, many of the methods developed are primarily for studies of Gram-negative bacteria, which are poorly suited to Gram-positive bacteria because of their dense peptidoglycan cell wall (Maharjan and Ferenci 2003). Many bacteria also have the capacity to form biofilms on a range of biological and abiotic substrates (O’Toole et al. 2000). Here, an extracellular matrix (ECM) encases bacteria, altering phenotype, biochemistry and transcriptome in comparison to free floating, planktonic, cells. Biofilms also alter the ability to extract metabolites from bacteria as well as making them recalcitrant to antimicrobial chemotherapy (Ramage et al. 2003; Drenkard and Ausubel 2002). Moreover, the transition from planktonic growth to biofilm formation can influence the metabolic profile of the bacteria (Gjersing et al. 2007). These factors make the metabolic analysis of clinically important pathogens problematic.

Metabolomics offers novel methods to study fundamental processes involved in microbial biofilm formation, their response to antimicrobial chemotherapy, and may also lead to the identification of novel biomarkers that could improve clinical diagnostics. To date, limited investigations have been performed on metabolomics of biofilms using nuclear magnetic resonance (NMR) spectrometry to study molecular differences between planktonic cells and biofilms (Zhang and Powers 2012; Ammons et al. 2014; Gjersing et al. 2007).

This paper aims to describe a novel method applicable to study biofilm metabolism in the model Gram-positive pathogen *Staphylococcus aureus*. Here, we report for the first time an optimised method for the extraction of the metabolome directly from a biofilm in parallel to cells living in suspension. Through liquid-chromatography-mass-spectrometry (LC–MS) metabolomics we demonstrate that the method is highly reproducible, further showing significant differences in metabolism between planktonic and biofilm cells grown under identical conditions. We focus on differences in arginine biosynthesis between the two growth states, thus providing insights into the biological properties of this important nosocomial pathogen.

## Materials and methods

All reagents and chemicals were purchased from Fisher Scientific, Loughborough, UK, at HPLC grade, unless stated otherwise.

### Bacteria culture

A clinical *S. aureus* strain, LHSKBClinical, (Stipetic et al. 2015), was used throughout this study. This was cultured on Brain Heart Infusion (BHI) agar plates or in BHI broth media (Oxoid, Basingstoke, UK). For all experiments, single colonies were taken from a plate and inoculated into liquid media. Cultures were grown overnight at 37 °C to stationary phase growth, which was used for subsequent planktonic and biofilm studies. Bacteria were grown under aerobic conditions.

### Planktonic cell preparation

For planktonic cell preparation, 200 µL of the stationary phase overnight culture were added to wells of a 96-well microtitre plate (Corning Incorporated, New York, USA), centrifuged at 1900×*g* at 4 °C for 3 min, and the spent media removed. Subsequently, 200 µl of 10 mM ammonium bicarbonate (Sigma-Aldrich, Dorset, UK) was added, the pellet resuspended, cells were washed by centrifugation at 1900×*g* at 4 °C for 3 min, and the supernatant was removed, providing a pellet for bead beating as described below.

### Biofilm cell preparation

Biofilms were prepared by adding standardised *S. aureus* culture at 1 x 10^8^ cells/ml in BHI into a 96-well microtitre plate, which was then statically incubated for 18 h at 37 °C under aerobic conditions. Following incubation, the supernatant was removed and the biofilms were washed in sterile 10 mM ammonium bicarbonate.

### Biofilm SEM

Scanning electron microscopy (SEM) was performed on parallel samples of *S. aureus* biofilms cultivated directly onto Thermanox^TM^ cover-slips (Nunc, Roskilde, Denmark), processed and imaged as previously described by Erlandsen et al. (2004).

### Metabolite extraction using bead beating

Metabolites were extracted directly within the wells of a microtitre plate by adding 200 µl of 0.1 mm acid washed, glass beads (Sigma-Aldrich) and ice cold (−20 °C) solvent solution (1 g of beads to 1 ml of chloroform: methanol:ddH_2_O [ratio of 1:3:1]).

A 96-well microtitre plate was used, primarily so that biofilms could be extracted in situ and so that 24 independent replicates could be performed for each condition, including media only negative controls. The plate was then sealed with a 96-well Cap Mat (Greiner Bio-One, Stonehouse, UK) and bead beaten on a cell disrupter (Disrupter Genie^®^) (Scientific industries, Inc., New York, USA), operating at a speed of 3000 RPM, at 4 °C continuously for 10 min. The plates were then centrifuged at 1900×*g* at 4 °C for 10 min, to remove beads and cell debris. The supernatant was then removed and stored at −80 °C until MS analysis. Extractions and metabolomics results represent one experiment with 24 replicates intended to demonstrate the utility of the method.

### Comparative methods for metabolite extractions

Two additional, previously published methods for Gram positive bacterial cell lysis (Soga et al. 2002; Soga et al. 2003; Takahashi et al. 2010), were compared to the bead beating method. Modifications to the published methods were made, and are described in detail in Online resource 1, but briefly, extraction solvents were altered to a chloroform/methanol/water 1:3:1 mix and water/PBS washes were replaced with 10 mM ammonium bicarbonate to ensure differences between the methods were due to lysis rather than extraction solvent. Sonication was altered from 30 s with a sonic probe to 30 min in a sonic bath to minimise heating and provide compatibility with microtitre plate extractions. Each extraction method was performed in triplicate.

### Metabolomic workflow, data acquirement and analysis

Samples were analysed by hydrophilic interaction liquid chromatography (HILIC) -mass spectrometry (LC–MS) (UltiMate 3000 RSLC (Thermo Fisher, San Jose, California, USA) using a 150 x 4.6 mm ZIC-pHILIC column (Merck SeQuant, Umea, Sweden) running at 300 µl/min and Orbitrap Exactive (Thermo Fisher) detection. Mass spectrometer parameters were: 50,000 resolving power in positive/negative switching mode. Electrospray ionisation (ESI) voltage was 4.5 kV in positive and 3 kV in negative modes. Buffers consisted of A: 20 mM ammonium carbonate in H_2_O and B: Merck SeQuant: acetonitrile. The gradient ran from 20 % A: 80 % B to 80 % A: 20 % B in 15 min, followed by a wash at 95 % A: 5 % B for 3 min, and equilibration at 20 % A: 80 % B for 5 min. Raw mass spectrometry data was processed using our standard pipeline, consisting of XCMS (Smith et al. 2006) (for peak picking), MzMatch (Scheltema et al. 2011) (for filtering and grouping) and in-house R-scripts (for further filtering, post-processing and identification). Peaks were visualised using PeakML Viewer (Scheltema et al. 2011). Core metabolite identifications were validated against a panel of unambiguous authentic pure standards using accurate mass and retention time (R_t_) and therefore could be classified using the alphanumeric metabolite coding scheme as ${\text{HRMS}}_{\text{a}}^{1}$ R_ta_ as described by Sumner et al. (2014). Additional putative identifications were assigned by accurate mass along with a R_t_ prediction algorithm (Creek et al. 2011) and therefore could be classified as ${\text{HRMS}}_{\text{PL}}^{1}$ (Sumner et al. 2014). Quantile normalisation was carried out across the data, normalising both data sets (Bolstad et al. 2003). Once identified and filtered, detected peak intensities were logged (base 2) and quantitation was performed on sets of biological replicates by applying differential statistics to generate P values. Metabolites with apparently different levels were assessed using Bayes moderated t-tests (Smyth 2004). Benjamini and Hochberg false discovery rate adjustment for multiple testing was applied (Hochberg and Benjamini 1990) and the resulting data was used to query KEGG (Kyoto Encyclopaedia of Genes and Genomes data base) for pathway analysis (Kanehisa and Goto 2000). Statistics, including principal component analysis (PCA), were performed and presented using R, employing appropriate standard R libraries and Microsoft Excel. ChemDraw Std version 14.0 was used to draw metabolic pathways and GraphPad Prism 4 used to create figures. Comparative metabolomic analysis of extraction methods was further processed using the IDEOM software (Creek et al. 2012), without normalisation applied.

## Results

### Liquid Chromatography-Mass Spectrometry metabolomics for *S. aureus* planktonic cell and biofilm analysis following sample bead-beating and metabolome extraction

**Fig. 1:**
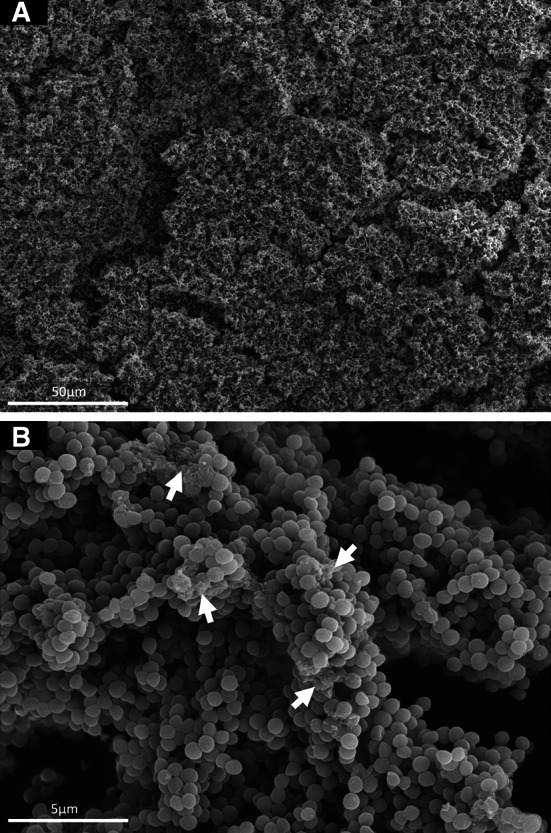
Scanning Electron Microscopy images of a clinical *S. aureus* strain biofilm, cultivated in BHI directly on Thermanox™ cover-slips for 18–20 h. **a** 500× magnification, **b** 5000× magnification. *Arrows* highlight areas of extracellular matrix

Static biofilms were grown using BHI, as an optimal medium based on a study of *S. aureus* biofilm formation in 1000 clinical isolates (Smith et al. 2008). SEM imaging (Fig. 1) provided qualitative data illustrating that the *S. aureus* clinically-derived strain used herein forms a biofilm. Figure 1 shows key characteristics of biofilm formation: cell clustering of cells attached and colonised to the surface of the cover-slip, and ECM production (O’Toole et al. 2000; Periasamy et al. 2012).

Once bacterial culture and biofilm cultivation had been established, an extraction platform was developed that would enable both planktonic and biofilm cells to be extracted simultaneously using a mechanical bead beating method.

A mechanical cell lysis method was optimised using 0.1 mm beads, as recommended for bacterial species by the cell disrupter manufacturer’s guidelines (Scientific industries 2014). Initial bead beating studies demonstrated that 10 min induced >95 % cell death (data not shown).

Greiner Bio-One Cap mats were found to be optimal for sealing plates during lysis, while the use of plastic-paraffin film resulted in sample leakage and cross contamination between samples. Ammonium bicarbonate was used for all wash stages due to its LC–MS compatibility and moderate pH buffering (Cassou et al. 2014; Faijes et al. 2007; Hedges et al. 2013). Extracting the metabolites at 4 °C in chloroform/methanol/water slowed (Laidler 1984) and quenched (t’Kindt and 2010) cellular metabolic activity during extraction.

A significant consideration for metabolomics is the amount of material (metabolite extraction) required for an effective analysis. Using the LC–MS set-up utilised here, a minimum sample volume of 10 µl of metabolite extraction with a minimum metabolite concentration dependent on the molecule studied, but detection limits generally in the micromolar range for small molecules (Gross 2011), were required for effective analysis. Sufficient material for detection and quantitation of hundreds of metabolites was obtained from a biofilm covering the well base of a 96-well microtitre plate.

### Comparison with alternative lysis methods

The extraction process described herein was compared to the methods described by Soga et al. (2002, 2003) and Takahashi et al. (Takahashi et al. 2010). No viable bacteria were found after analysis of the pellet using any of the described methods (data not shown). Total useable signal of the metabolites from each method showed no significant difference between the lysis methods (*t* test P values of 0.12—bead beating vs. filtration, 0.10—bead beating vs. sonication for positive mode and 0.15—bead beating vs. filtration, 0.09 bead beating vs. sonication for negative mode data) (see Online resource 2). Further analysis of the extractions performed using the filtration method, however, demonstrated a general decrease in the intensities of well characterised metabolites. Of 84 ‘identified’ metabolites, 31 are significantly lower in intensity with only 3 significantly higher, while the bead beating and sonication methods gave similar results overall (see Online resource 2).

### Liquid-chromatography-mass spectrometry (LC–MS) data analysis

Data analysis of cell extractions derived from planktonic shaking cultures compared to static biofilm extractions yielded 530 significantly changing peaks with an adjusted P value of <0.05 (null hypothesis: there is no difference between planktonic cell and biofilm extractions, reject null hypothesis). Of these, 151 metabolites demonstrated a significant up-regulation in expression in planktonic cells compared to biofilms, with log_2_ fold changes of ≥1. Conversely, 177 were found to be significantly up-regulated in biofilms as compared to planktonic cells with log_2_ fold changes of ≥1. Metabolites were identified by matching mass and R_t_ to internal standards or annotated by mass-matching to a database entry. Data analyses, including raw peak data, metabolite identification, planktonic compared to biofilm samples analysis, and R_t_ errors are shown in Online Resource 3.

Through plotting the average peak intensities between replicates of peaks listed in Table 1 (Online Resource 4), demonstrates that the deviation between replicates in the sample group is relatively small, and highlights differences in the fold changes between sample groups. All individual peak intensities are shown in Online Resource 3, raw data.

**Table 1:** Arginine Biosynthesis metabolites identified from planktonic and biofilm data sets

Peak number	Metabolite	Elemental formula	KEGG ID^a^	Metabolite code^b^	Mass [M–H] (Da)	R_t_^c^ (sec)	Log_2_ fold change^d^
2782	Aspartate	C_4_H_7_NO_4_	C00049	${\text{HRMS}}_{\text{PL}}^{1}$	132.0302	749.1	1.577
2873	Glutamate	C_5_H_9_NO_4_	C00025	${\text{HRMS}}_{\text{a}}^{1}$ R_ta_	146.0458	725.7	1.0159
2981	Glutamine	C_5_H_10_N_2_O_3_	C00064	${\text{HRMS}}_{\text{a}}^{1}$ R_ta_	145.0618	781.3	n/a^(e)^
3171	Citrulline	C_6_H_13_N_3_O_3_	C00327	${\text{HRMS}}_{\text{a}}^{1}$ R_ta_	174.0884	813.1	3.5413
3252	N-Acetyl-l-glutamate	C_7_H_11_NO_5_	C00624	${\text{HRMS}}_{\text{a}}^{1}$ R_ta_	188.0564	691.1	4.3654
3505	l-Arginosuccinate	C_10_H_18_N_4_O_6_	C03406	${\text{HRMS}}_{\text{PL}}^{1}$	289.1156	800.5	n/a^f^
3608	N-Acetyl-l-citrulline	C_8_H_15_N_3_O_4_	C15532	${\text{HRMS}}_{\text{PL}}^{1}$	216.099	594.1	5.049
3636	N-Acetyl-ornithine	C_7_H_14_N_2_O_3_	C00437	${\text{HRMS}}_{\text{a}}^{1}$ R_ta_	173.0931	791.2	1.068
3838	Arginine	C_6_H_14_N_4_O_2_	C00062	${\text{HRMS}}_{\text{PL}}^{1}$	173.1044	1304.1	1.3781

^a^See reference Kanehisa and Goto 2000

^b^See reference Sumner et al. 2014

^c^Retention time

^d^Log_2_ fold change in expression of metabolite between planktonic and biofilm data sets

^e^Not available, fold change unable to be formulated as peak intensities did not significantly change between sample sets

^f^Not available, fold change unable to be formulated as only detected in the planktonic data set and not in the biofilm data set

**Fig. 2:**
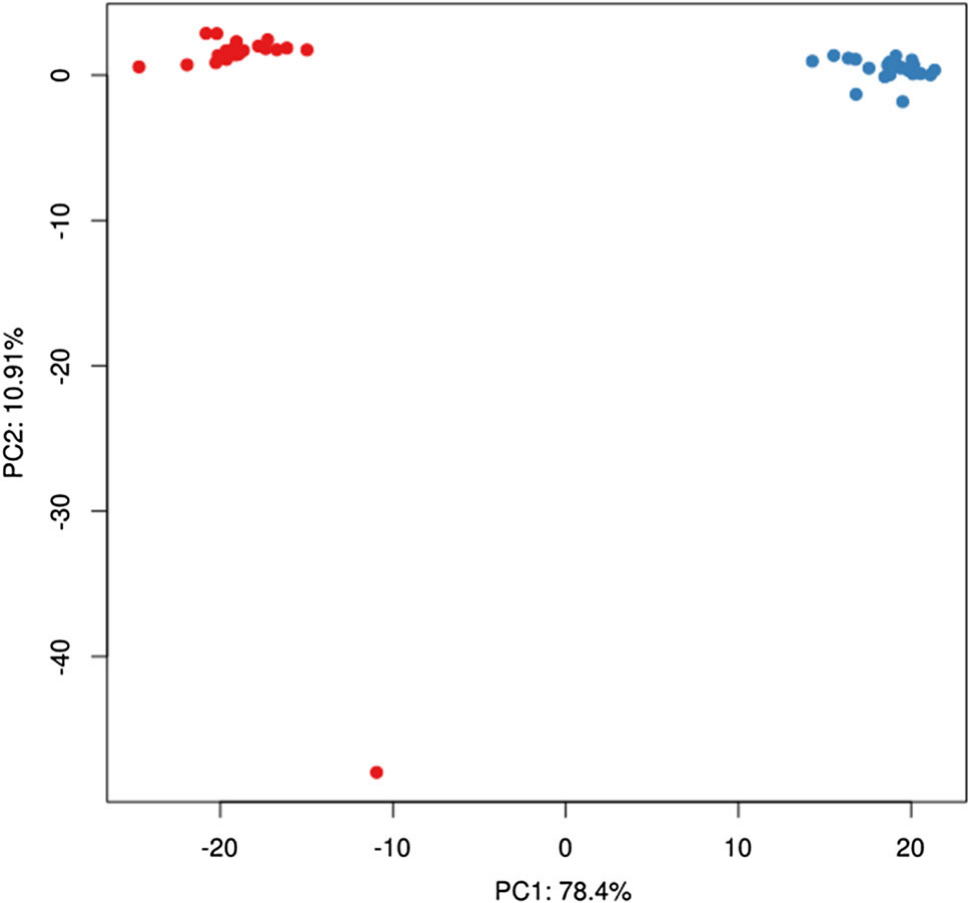
Principal component analysis (PCA) plots of Planktonic (*blue*) and biofilm (*red*) metabolomic data sets, utilising a 10 min bead beating extraction method; beads in a 50:50 suspension in extraction solvent of chloroform; methanol; water (ratio 1:3:1), followed by liquid-chromatography-mass spectrometry. *Red* and *blue* data plots represent planktonic cells and biofilm biological sample replicates, respectively. n = 24 (Color figure online)

PCA of metabolomic data sets of planktonic cells and biofilm samples, analysed using LC–MS, revealed clustering of biological replicates (Fig. 2). The plot shows variation in detected metabolites between both groups. Principal components 1 and 2 are responsible for 78.4 and 10.91 % of the variation, respectively. Furthermore, the plot shows an outlier in the biofilm data set not clustered with the other replicates along PCA 2. This sample displays low or zero peak intensities in most metabolites compared to other replicates in the set (see Online Resource 3, raw data, sample B20) and is likely to be due to a failed injection.

### Arginine Metabolism- an example of a metabolic pathway showing significant changes between planktonic and biofilm samples

Metabolites were matched to pre-existing pathways defined by the KEGG database (Kanehisa and Goto 2000). The results yielded 129 pathways containing a minimum of two identified (${\text{HRMS}}_{\text{a}}^{1}$ R_ta_) or annotated (${\text{HRMS}}_{\text{PL}}^{1}$) metabolites that demonstrated significant (P < 0.05) changes in expression between planktonic cells and biofilm samples. Table 2 lists the top 20 of these pathways annotated with the most detected metabolites. All further identified and annotated compounds grouped into pathways are listed in Online Resource 3.

**Table 2:** Top 20 metabolic pathways that have intermediate and end-product metabolites that display significant changes in intensity between planktonic cells and biofilm samples

Pathway name^a^	KEGG map ID^a^	Number of metabolites^b^	Annotated (${\text{HRMS}}_{\text{PL}}^{1}$)^c^	Identified (${\text{HRMS}}_{\text{a}}^{1}$ R_ta_)^d^	Coverage^e^ (%)	P vs. B^f^
Arginine and proline metabolism/arginine biosynthesis	00330/00220	90	49	5	60	33
Protein digestion and absorption	04974	47	22	7	61.7	19
Tyrosine metabolism	00350	76	35	2	48.7	18
Histidine metabolism	00340	45	25	3	62.2	17
Galactose metabolism	00052	41	22	0	53.7	17
Aminoacyl-tRNA biosynthesis	00970	52	13	7	38.5	17
Cyanoamino acid metabolism	00460	46	31	1	69.6	16
Linoleic acid metabolism	00591	28	26	0	92.9	15
Limonene and pinene degradation	00903	64	53	0	82.8	15
C5-Branched dibasic acid metabolism	00660	32	20	4	75	14
Mineral absorption	04978	29	11	4	51.7	14
Phosphotransferase system (PTS)	02060	48	20	1	43.8	14
Two-component system	02020	41	10	6	39	14
Fructose and mannose metabolism	00051	51	16	0	31.4	14
Alanine, aspartate and glutamate metabolism	00250	24	15	2	70.8	13
Lysine degradation	00310	47	26	1	57.4	13
Glycine, serine and threonine metabolism	00260	51	27	2	56.9	13
Phenylalanine metabolism	00360	72	33	1	47.2	13
Pyrimidine metabolism	00240	66	28	5	50	12
Aminobenzoate degradation	00627	84	28	1	34.5	12
Purine metabolism	00230	92	16	9	27.2	12
Citrate cycle (TCA cycle)	00020	20	5	3	40	4

^a^In accordance with KEGG (Kyoto Encyclopaedia of Genes and Genomes data base) (Kanehisa and Goto 2000)

^b^Total number of metabolites in the pathway according to KEGG

^c^Number of annotated metabolites detected, metabolite code ${\text{HRMS}}_{\text{PL}}^{1}$ (Sumner et al. 2014)

^d^Number of identified metabolites detected, metabolite code ${\text{HRMS}}_{\text{a}}^{1}$ R_ta_ (Sumner et al. 2014)

^e^Percentage of metabolites detected in the pathway (${\text{HRMS}}_{\text{PL}}^{1}$ and ${\text{HRMS}}_{\text{a}}^{1}$ R_ta_)

^f^Number of metabolites detected (${\text{HRMS}}_{\text{PL}}^{1}$ and ${\text{HRMS}}_{\text{a}}^{1}$ R_ta_) that show significant changes in expression profiles between Planktonic (P) and biofilm (B) samples

**Fig. 3:**
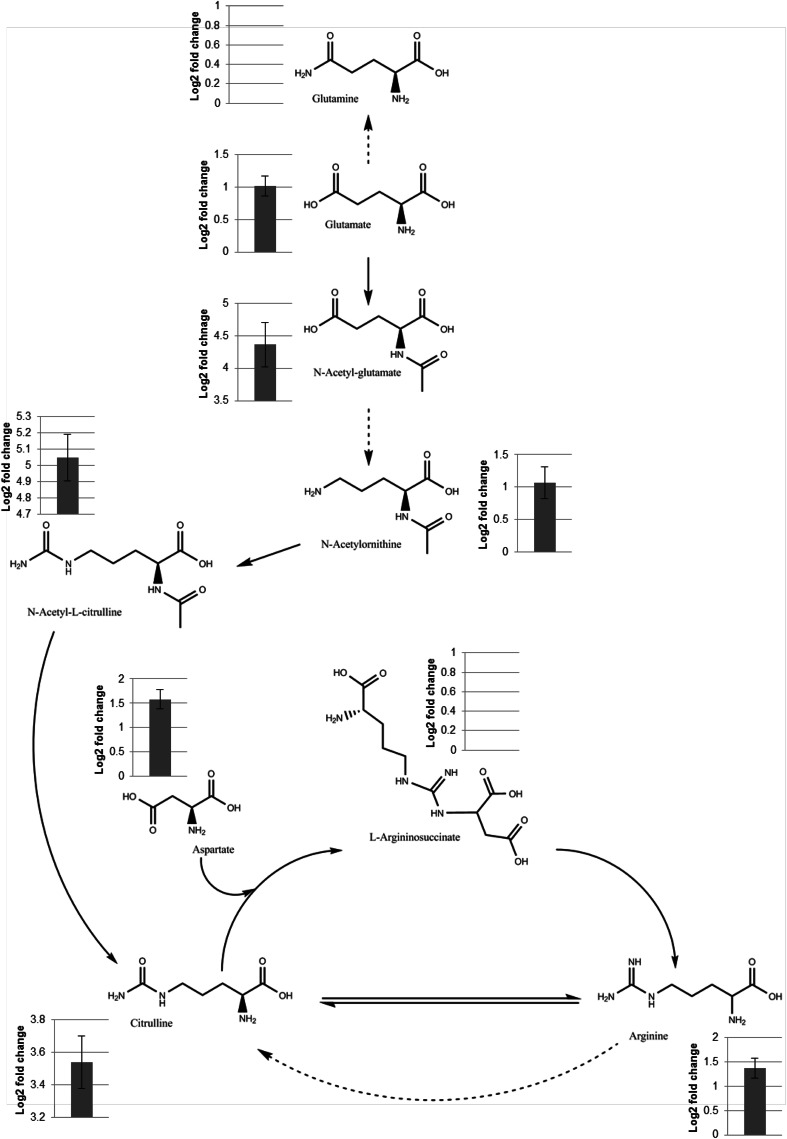
Arginine biosynthesis metabolic pathway showing both detected metabolites identified by having mass and retention time (R_t_) matched to an internal standard (*Bold* and *underlined*) and annotated metabolites matched to an accurate mass database entry. Full molecular structures of the metabolites are given. *Graphs* represent Log_2_ fold change of metabolite intensity/expression in planktonic cells compared to biofilms. A positive fold change indicates expression was either up regulated in planktonic samples or down regulated in biofilm samples. Pathway and structures annotated from the Kyoto Encyclopedia of Genes and Genomes (KEGG) database. *Block arrows* represent a direct link between metabolites. *Dashed arrows* represent the presence of metabolite intermediates that were not detected. *Error bars* represent 99.95 % fold change confidence interval

After mapping the data to arginine biosynthesis (KEGG pathway 00220), with filtering based on the available genome sequence of this isolate, it was found that 4 identified (${\text{HRMS}}_{\text{a}}^{1}$ R_ta_) and 5 putatively annotated (${\text{HRMS}}_{\text{PL}}^{1}$) (total of 9) metabolites are involved in this pathway in *S. aureus* (Table 1 and Fig. 3). Of these, 6 metabolites (2 identified and 4 annotated) were seen to have significant (P < 0.05) log_2_ fold changes between planktonic and biofilm sample sets. Peak annotation results of the 9 identified or annotated arginine biosynthesis metabolites detected in negative mode are listed in Table 1. A number of metabolites were identified or annotated in both data sets were a fold-change difference in intensity could be calculated (Table 1), however L-arginosuccinate was detected in the planktonic data set, displaying a peak in all sample replicates, but not detected in the biofilm data set. Glutamine was identified in both planktonic and biofilm data sets, however its peak intensities did not significantly change between sample sets. Peak intensity graphs for the peaks listed in Table 1 are shown in Online Resource 5.

## Discussion

Most bacteria possess the ability to live in suspension or as an adhered community on a surface. Novel ways to study the distinct differences in metabolism between these two states are much needed so that differences can be exploited with the development of new antibiotics, in addition to understanding fundamental biological processes. Further to this, studying Gram-positive bacteria and their intracellular metabolic intermediates comes with additional complications due to the robustness of the thick peptidoglycan cell wall. Data presented herein describes a novel metabolite extraction method utilising a direct bead beating method for biofilms cultivated in a 96-well format, and down-stream analysis to compare planktonic and biofilm cells. Many previously published approaches for biofilm investigations use 96-well plates, allowing the bacterial culture to adhere and develop on the bottom of the well (O’Toole 2011; Merritt et al. 2005; Christensen et al. 1985). The use of a 96-well plate format has the additional benefit of allowing for the substantial multiplexing of samples on the same plate, as previously shown (Pierce et al. 2008; Lopez-Ribot 2014; Srinivasan et al. 2013), including metabolite analysis. Of note is that current published metabolite extraction methods using bead beating focus on bacterial cells living in a planktonic state (Liebeke et al. 2012). This method has previously been shown to be beneficial in the extraction of DNA and metabolites from Gram-positive bacteria (Liebeke et al. 2012), pathogenic yeasts (Bolano et al. 2001) and filamentous fungi (van Burik et al. 1998); organisms that also possess a thick cell wall.

We were unable to use standard enzymatic disruption methods (Salazar 2007), as this would introduce the requirement for ambient temperature resulting in metabolite degradation. However, we compared lysis using bead beating with two other methods, filtration followed by solvent extraction alone (2002; 2003), and sonication (Takahashi et al. 2010). Solvent extraction was demonstrated to be less effective than either bead beating or sonication, while sonication was comparable to bead beating, giving broadly similar results, so can be considered equally viable for analysis of planktonic Gram-positive bacteria. The limitation of sonication for biofilm experiments include the complexities of sonicating uniformly across a multiwell plate and the time taken for bath sonication methods.

Previous metabolomic literature has reported a number of different solvent mixtures, demonstrating that the solvent used and the temperature of extraction determines the diversity of extracted metabolites (A et al. 2005). We used chloroform methanol water 1:3:1 as an optimal extraction solvent for our chromatography system (t’Kindt et al. 2010). A practical concern with using solvents such as chloroform in cell-culture plastics is they can etch and degrade plastic (Bawn and Wajid 1956). To avoid this, glass coated wells and glass cell culture materials can be used. However, previous work has demonstrated that biofilm formation is altered on different substrates (Jansen and Kohnen 1995; Ramage et al. 2003; Passerini de Rossi et al. 2007), so the use of plastic is often unavoidable. It was found here that the effects of plastic degradation on overall metabolomic results was minimal, possibly due to the poor retention of hydrophobic compounds on the pHILIC column used, and if present could be compensated for with the use of an appropriate negative control. In addition, any plastic debris would also be pelleted along with the beads at the end of the extraction process, and may be discarded as the supernatant contains the metabolite extract.

One metabolic pathway that shows significant changes is arginine metabolism, specifically the urea cycle (Fig. 3). Several pathway intermediates: glutamate (${\text{HRMS}}_{\text{a}}^{1}$ R_ta_); N-acetyl-l-glutamate (${\text{HRMS}}_{\text{a}}^{1}$ R_ta_); N-acetyl-l-citrulline (${\text{HRMS}}_{\text{PL}}^{1}$); aspartate (${\text{HRMS}}_{\text{PL}}^{1}$); citrulline (${\text{HRMS}}_{\text{a}}^{1}$ R_ta_); arginine (${\text{HRMS}}_{\text{PL}}^{1}$); and N-acetyl-ornithine (${\text{HRMS}}_{\text{a}}^{1}$ R_ta_) were detected to be significantly up-regulated in planktonic samples or down-regulated in the biofilm samples, suggesting that they are depleted in response to rapid flux through this pathway. These results correlate with previous studies that show significant changes in energy and cell metabolism between planktonic cells and biofilms (Zhang and Powers 2012; Ammons et al. 2014; Gjersing et al. 2007). Previous work using microarrays also showed up-regulation of urea cycle genes in response to the formation of biofilms in *S. aureus* (Resch et al. 2005). The amino acid glutamine was identified (${\text{HRMS}}_{\text{a}}^{1}$ R_ta_) in both data sets but was seen not to be changing in intensity between planktonic and biofilm samples. *S. aureus* selectively extracts the amino acid glutamine from its medium environment (Zhu et al. 2007). The metabolite l-arginosuccinate was annotated (${\text{HRMS}}_{\text{PL}}^{1}$) to be found in planktonic samples only and was absent from biofilm data. The absence in biofilms may be because the metabolite is simply not present or because its concentration is below the limits of detection of our instrumentation. Previous studies by Zhu et al. (2007) and Ammons et al. (2014) suggest that changes in amino acid metabolism are a key feature differing in biofilms compared to planktonic samples. Furthermore, these studies and Wu et al. (2010) suggest that arginine metabolism and catabolism play an important role in biofilm survival. We hypothesise that *S. aureus* may amplify flux through the urea cycle to generate ammonia to restore pH balance in response to the production of acid in the biofilm ECM (Resch et al. 2005) due to anaerobic glycolysis. This correlates with other studies that suggest that amino acid catabolism is crucial for biofilm pH balance (Beenken et al. 2004; Resch et al. 2006; Resch et al. 2005; Zhu et al. 2007).

This study highlights key metabolic differences in arginine metabolism between different bacterial growth states. Findings presented here show that metabolism is significantly altered by the same species of bacteria once a sessile growth phase has been initiated. The changes identified here between growth states could be evidence of the bacteria responding to their changing environment and trying to maintain a ‘status quo’ in chemical and pH balance, Bacteria should be thought of as dynamic entities capable of displaying significantly altered phenotypes without the necessity of genetic change. Because of this, research into bacteria, and especially in the field of antimicrobial testing and identification, should consider different effects drugs may have on different stages of growth.

### Method limitations

The biofilm cultivation method presented here in a 96-well format, is based on a static growing biofilm. It is known that biofilm phenotype can change depending on whether cultivated in a static or flow system (Weaver et al. 2012; Yarwood et al. 2004). However, the bead-beating extraction method described here can be applied to biofilms cultivated on cover-slips used in a flow system, whereby the cover-slip with attached biofilm is removed, washed and placed in a bijou or similar for extraction. The extraction method also does not discriminate between the biofilm encased cells and the ECM. An appropriate ECM and cell extraction method, suitable for mass-spectrometry metabolomic samples is a target for future method development. Finally, due to the resilience of the cells and the time taken for full lysis and extraction, rapid metabolic processes may still continue under these conditions, and should be taken into account when evaluating the results.

## Conclusions

The low-temperature, rapid, mechanical method coupled with an extraction solvent to lyse the peptidoglycan cell wall and extract the cellular metabolome from Gram-positive cells living in a planktonic state or as a biofilm is described here. The method is shown to be a highly reproducible platform to study cellular metabolism. PCA clustering of 24 replicates, low variation in average peak intensities and identification of significant (adjusted P < 0.05) changes in abundance of metabolite components of the arginine biosynthesis pathway between data sets highlights this reproducibility. Comparison with other methods for lysis of planktonic bacteria further demonstrates its utility for general bacterial metabolomics.

Data shows that significant changes in a number of metabolic pathways, highlighting arginine biosynthesis, take place between planktonic cells and biofilms. During static culture, the biofilm’s adherence to the base of the well will necessarily result in reduced access to oxygen, in contrast with a planktonic, shaking culture, and this change in oxygen availability may be responsible for some or many of the changes observed. However, is a general consensus that metabolism is significantly altered in biofilm-forming cells compared to planktonic cells (Zhang and Powers 2012; Resch et al. 2006; Gjersing et al. 2007). The study of microbial metabolism is challenging but provides crucial insight into the biochemistry of bacteria. The added complication of bacterial physiology responding to its environment adds to the complexity of such studies. The ability to study metabolism provides the potential for inferences to be gained, such as the modes of action of antimicrobials and the identification of new metabolic drug targets. The insurgence of antimicrobial resistance (Arias and Murray 2015) means that research in this area is vitally important.

## Electronic supplementary material

Supplementary material 1 (DOCX 18 kb)

Supplementary material 2 (XLSM 37155 kb)

Supplementary material 3 (XLSX 5015 kb)

Supplementary material 4 (DOCX 303 kb)

Supplementary material 5 (DOCX 389 kb)
