# Supplementary material for: A novel metabolomic approach used for the comparison of Staphylococcus aureus planktonic cells and biofilm samples
Source: Metabolomics. 2016 Mar 8;12:75. doi: 10.1007/s11306-016-1002-0 (PMC4783440; doi:10.1007/s11306-016-1002-0)
Supplement: Supplementary file 1 — Supplementary material 1 (DOCX 18 kb) [file 11306_2016_1002_MOESM1_ESM.docx]

**ESM_1: Supplementary methods.**

*Comparative methods for metabolite extractions*

Bead beating was performed as described in the main methods section except that lysis was performed in 1.5 ml reaction tubes rather than microtitre plates to maintain consistency in volume and process with comparative methods.

Previously published methods for Gram positive bacterial lysis

Filter method adapted from Soga et al. 2002, Soga et al. 2003

10 ml planktonic stationary phase cell culture was passed through a 0.45 µm pore size Whatman^TM^ filter (Sigma-Aldrich) using a Nalgene 250 ml filter unit (Thermo-scientific) attached to a vacuum pump. Cells on the filter were then washed with 10 ml of 10 mM ammonium bicarbonate (Sigma-Aldrich). Following washing, the filter was removed and placed in a 15 ml falcon tube (Corning) where 5 ml of 10 mM ammonium bicarbonate was used to wash and detach cells from the filter. Cells were then centrifuged at 1900 g for 5 mins at 4°C, discarding the supernatant. The cell pellet was resuspended in 1 ml ice cold metabolite extraction solvent mix consisting of chloroform:methanol:ddH_2_O at a ratio of 1:3:1, described by T’kindt et al. (T’Kindt et al. 2010), transferred to a 1.5 ml reaction tube and incubated at room temperature, shaking, for 5 mins.

Sonication method adapted from Takahashi et al. 2010

10 ml planktonic stationary phase cells were washed twice in 10 mM ammonium bicarbonate by centrifugation at 1900 g for 5 mins at 4 °C. Cells were then resuspended in 1 ml of ice cold metabolite extraction solvent mix, as described above, in 1.5 ml reaction tubes. Samples were then sonicated for 30 mins in a Grant XUB5 sonication bath (Grant, Shepreth, UK) operating at a frequency of 38 kHz, filled with ice and water, and then vortexed briefly.
