# Supplementary material for: A novel metabolomic approach used for the comparison of Staphylococcus aureus planktonic cells and biofilm samples
Source: Metabolomics. 2016 Mar 8;12:75. doi: 10.1007/s11306-016-1002-0 (PMC4783440; doi:10.1007/s11306-016-1002-0)
Supplement: Supplementary file 4 — Supplementary material 4 (DOCX 303 kb) [file 11306_2016_1002_MOESM4_ESM.docx]

**ESM_4: Supplementary Figure, Arginine biosynthesis metabolites** showing average peak intensities with standard deviation error between replicates of planktonic and biofilm sample sets.

**
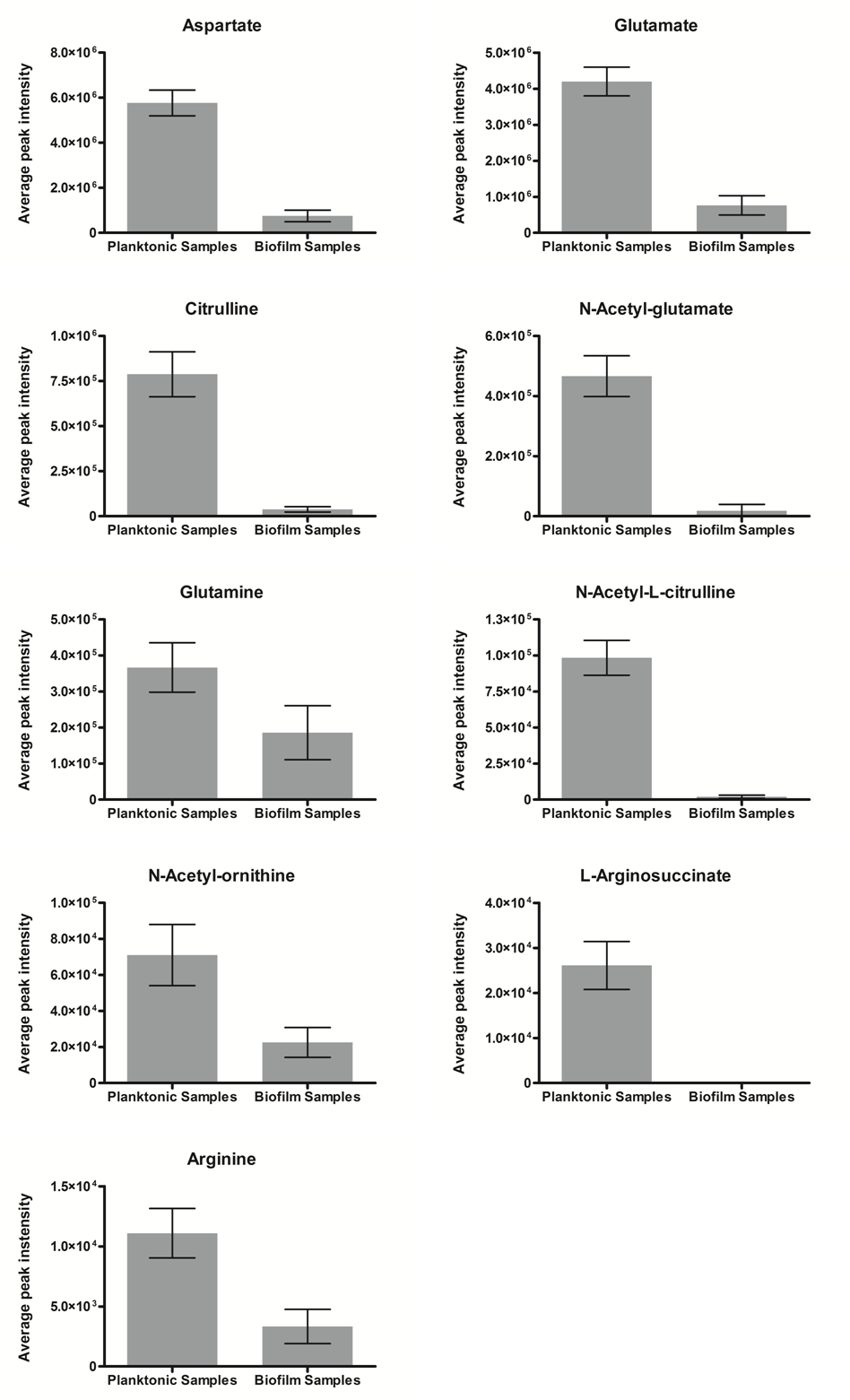
**
