# Supplementary material for: A novel metabolomic approach used for the comparison of Staphylococcus aureus planktonic cells and biofilm samples
Source: Metabolomics. 2016 Mar 8;12:75. doi: 10.1007/s11306-016-1002-0 (PMC4783440; doi:10.1007/s11306-016-1002-0)

**ESM_5: Supplementary Figure, Peak intensity graphs for Arginine biosynthesis metabolites** showing each replicate peak intensity. Created and visualised using Peak ML viewer. Red peaks represent biofilm replicates and purple peaks represent planktonic replicates.


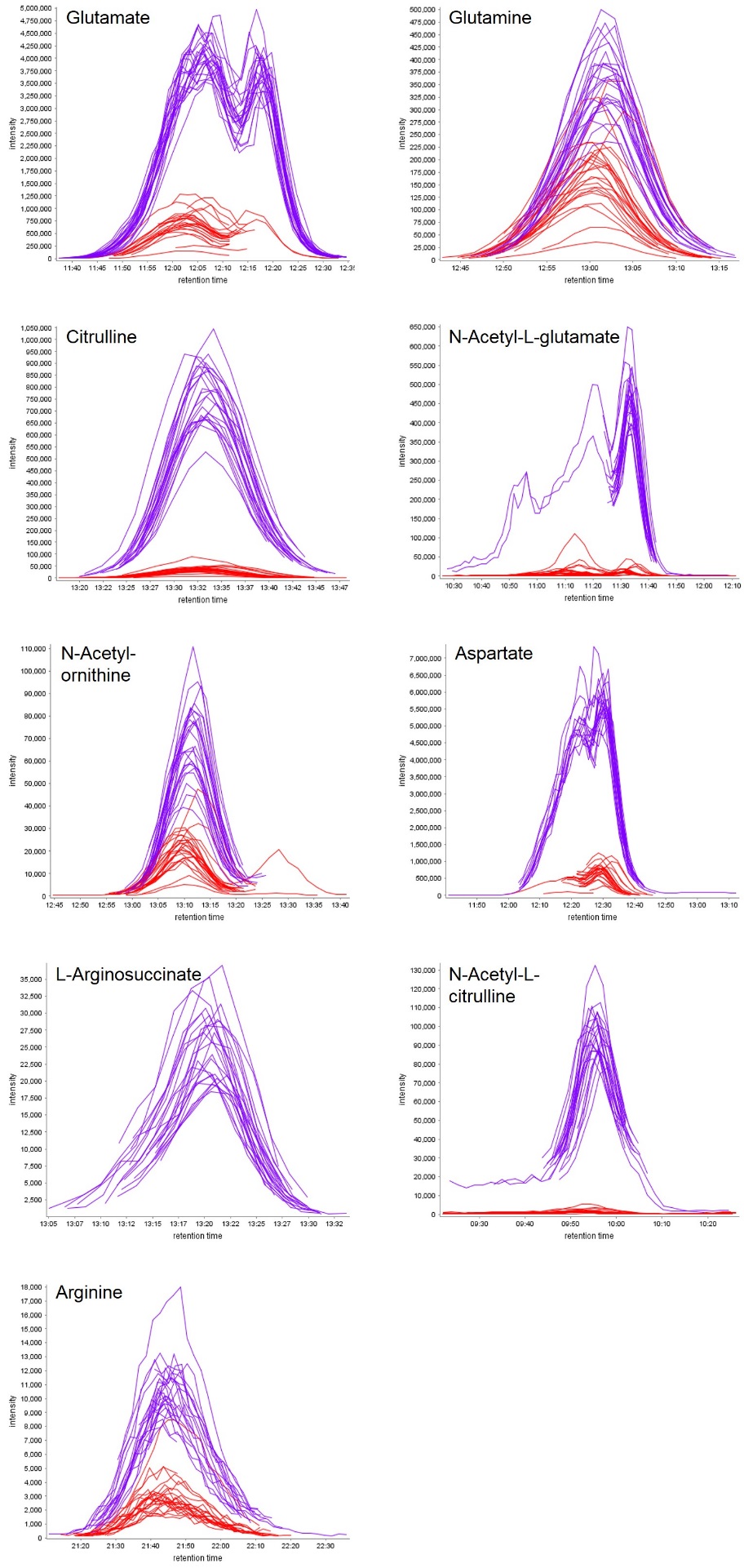

Supplement: Supplementary file 5 — Supplementary material 5 (DOCX 389 kb) [file 11306_2016_1002_MOESM5_ESM.docx]
